# Culture Media and Individual Hosts Affect the Recovery of Culturable Bacterial Diversity from Amphibian Skin

**DOI:** 10.3389/fmicb.2017.01574

**Published:** 2017-08-24

**Authors:** Daniel Medina, Jenifer B. Walke, Zachary Gajewski, Matthew H. Becker, Meredith C. Swartwout, Lisa K. Belden

**Affiliations:** Department of Biological Sciences, Virginia Tech Blacksburg, VA, United States

**Keywords:** amphibian, amphibian skin bacteria, *Batrachochytrium dendrobatidis*, low nutrient culture media, microbiome, R2A

## Abstract

One current challenge in microbial ecology is elucidating the functional roles of the large diversity of free-living and host-associated bacteria identified by culture-independent molecular methods. Importantly, the characterization of this immense bacterial diversity will likely require merging data from culture-independent approaches with work on bacterial isolates in culture. Amphibian skin bacterial communities have become a recent focus of work in host-associated microbial systems due to the potential role of these skin bacteria in host defense against the pathogenic fungus *Batrachochytrium dendrobatidis* (Bd), which is associated with global amphibian population declines and extinctions. As there is evidence that some skin bacteria may inhibit growth of Bd and prevent infection in some cases, there is interest in using these bacteria as probiotic therapy for conservation of at-risk amphibians. In this study, we used skin swabs from American toads (*Anaxyrus americanus*) to: (1) assess the diversity and community structure of culturable amphibian skin bacteria grown on high and low nutrient culture media, (2) determine which culture media recover the highest proportion of the total skin bacterial community of individual toads relative to culture-independent data, and (3) assess whether the plated communities from the distinct media types vary in their ability to inhibit Bd growth in *in-vitro* assays. Overall, we found that culture media with low nutrient concentrations facilitated the growth of more diverse bacterial taxa and grew distinct communities relative to media with higher nutrient concentrations. Use of low nutrient media also resulted in culturing proportionally more of the bacterial diversity on individual toads relative to the overall community defined using culture-independent methods. However, while there were differences in diversity among media types, the variation among individual hosts was greater than variation among media types, suggesting that swabbing more individuals in a population is the best way to maximize culture collections, regardless of media type. Lastly, the function of the plated communities against Bd did not vary across culture media type or between high and low nutrient media. These results inform current efforts for developing a probiotic-based approach for amphibian conservation and help to ensure that culture collections are capturing the majority of the important diversity in these systems.

## Introduction

Microbial ecologists currently face the challenge of characterizing the ecological role of the vast bacterial diversity associated with different environments and hosts, identified by culture-independent methods, such as 16S rRNA gene amplicon sequencing (Zengler et al., 2002; Vartoukian et al., 2010). A complete understanding of the metabolic, pathogenic and functional features of this diversity will be enhanced by the use of “-omics” methods, such as shotgun metagenomics, the cultivation and isolation of the bacterial species (Zengler et al., 2002) and by linking culture-independent and culture-dependent approaches (Rebollar et al., 2016a). Importantly, culturable bacteria have provided a good system for assessing ecological processes (Jessup et al., 2005). For instance, bacterial systems have been used to assess the generality of some key factors known to influence and determine diversity patterns in plants and animals, such as habitat type, habitat heterogeneity, disturbance and primary productivity (reviewed in Horner-Devine et al., 2004). In addition, expanding our understanding about the natural history of bacteria, which are essential in critical natural processes, such as nutrient cycling, requires at some level the use of cultured isolates (Jessup et al., 2004). Lastly, the cultivation of bacteria can play a key role in the development of probiotics to mitigate pathogen infection in an array of species (Bletz et al., 2013; Walke and Belden, 2016). While “-omics” approaches can assist with identification of potential probiotics, implementation will still require the cultivation of bacteria for biological assays and probiotic application (e.g., Rebollar et al., 2016a).

Bacterial communities associated with amphibian skin have received attention due to the potential defensive role they play against the amphibian fungal skin pathogen *Batrachochytrium dendrobatidis* (Bd) (Bletz et al., 2013; Walke and Belden, 2016). The potential defensive role of the amphibian skin bacteria is important from an ecological and conservation standpoint given that chytridiomycosis, the disease caused by Bd, has been associated with amphibian extinctions and population declines around the world (Berger et al., 1998; Bosch and Martínez-Solano, 2006; Lips et al., 2006; Crawford et al., 2010; Vredenburg et al., 2010), and has caused a substantial disease-related loss of biodiversity (Fisher et al., 2012; Lips, 2016). Amphibian skin bacterial communities might also serve as a microbial system to address fundamental ecological questions, in particular those focused on the processes influencing diversity, community assembly, and function. For example, amphibian skin bacterial communities have been used to determine the roles of neutral (e.g., dispersal and ecological drift) and deterministic processes (e.g., habitat filtering and competition) in shaping bacterial communities (Loudon et al., 2016), to assess the influence and feedbacks caused by an invasive species, such as a host skin pathogen (Jani and Briggs, 2014), to examine the relationship between community structure and function (Becker et al., 2015; Belden et al., 2015; Walke et al., 2015b), and to elucidate the factors potentially driving context-dependent function (Daskin et al., 2014; Loudon et al., 2014; Woodhams et al., 2014).

The use of culture-dependent techniques has played an important role in the study of bacteria associated with amphibian skin, mainly within a conservation context to assist in the development of a probiotic-based conservation method. Culture-dependent techniques have facilitated the development of protocols to identify skin bacterial isolates that inhibit Bd growth *in vitro* (e.g., Harris et al., 2006; Lauer et al., 2007, 2008; Flechas et al., 2012; Bell et al., 2013; Becker et al., 2015), and also to identify some of the ecological factors (e.g., interspecific competition and temperature) affecting the production of bacterially-produced secondary metabolites (Daskin et al., 2014; Loudon et al., 2014).

In ecology, studies manipulating nutrient concentration and composition have advanced understanding of the relationship between primary productivity and diversity in terrestrial plant communities (Goldberg and Miller, 1990; Wilson and Tilman, 1991), aquatic communities (Schindler, 1990) and microbial systems (Kassen et al., 2000). Within this context, when cultivating bacteria, the composition and diversity of the cultured community is limited by factors, such as incubation time and culture media nutrient concentration and composition (Stevenson et al., 2004). Thus, nutrient composition and availability can influence bacterial communities and enhance the cultivation of previously uncultured bacteria (Vartoukian et al., 2010). For instance, nutrient-rich culture media (i.e., complex media) favors the growth of fast-growing bacteria over slower-growing bacteria (Connon and Giovannoni, 2002; Vartoukian et al., 2010), and the dilution of nutrients in culture media has been used to cultivate previously uncultured bacteria (Connon and Giovannoni, 2002; Zengler et al., 2002; Vartoukian et al., 2010).

In this study, we aimed, from an ecological perspective, to assess how high and low nutrient culture media influence the diversity of cultured amphibian skin bacteria that are recovered. In addition, and within an applied context, we also assessed the ability of different culture media types to grow a high portion (i.e., number of OTUs) of amphibian skin bacteria, and determined what culture media can recover the most representative fraction of the bacterial community relative to a culture-independent method. Lastly, given the ability of some bacterial isolates to inhibit Bd growth *in vitro*, particularly when grown with other bacteria (Loudon et al., 2014), and their potential as a conservation approach to mitigate Bd infection, we aimed to determine whether the bacterial communities growing on the different media types differed in their ability to inhibit Bd growth.

## Materials and methods

### Sample collection

We analyzed bacterial communities from skin swabs from 12 American toads (*Anaxyrus americanus)* collected in Jefferson National Forest near Blacksburg, VA (USA). In the field, toad skin bacterial communities were sampled as described by Walke et al. (2015a). Briefly, each individual toad was handled with a new pair of nitrile gloves, rinsed twice with sterile water to remove transient microbes (Walke et al., 2014; Belden et al., 2015), and swabbed sequentially with two sterile rayon swabs (MW113; Medical Wire & Equipment). The swabbing technique was standardized and consisted of 20 strokes on the ventral side of the toad and 5 strokes along each thigh and foot. The first swab was placed in a 1.5 ml sterile microcentrifuge tube and was used to characterize the skin bacterial communities via culture-independent 16S rRNA gene amplicon sequencing. The second swab was stored in another 1.5 ml sterile microcentrifuge tube containing 100 μl of TSYE-glycerol medium (2% Trypticase soy broth, 1% yeast extract, 20% glycerol) and was used for the characterization of bacterial isolate communities grown on four different types of culture media. These cultured communities were also subsequently characterized via 16S rRNA gene amplicon sequencing and were tested for whole community *in vitro* inhibition of Bd growth. Both field-collected swabs were stored at −80C until processing. All animal use was approved by the Institutional Animal Care and Use Committees of Virginia Tech.

### Culture-independent assessment of the skin bacterial communities

DNA was extracted from the first swab using the Qiagen DNeasy blood and tissue kit (Valencia, CA) protocol for Gram positive bacteria, with an initial incubation step of 1 h at 37°C. For the community characterization, the V4 region of the 16S rRNA gene was amplified using the primers 515F and the barcoded 806R and sequenced using a 250 bp paired-end strategy on the Illumina MiSeq platform as described by Walke et al. (2015a). The culture-independent data produced and used in the present study was part of Walke et al. (2015a), and is available in NCBI's Sequence Read Archive (SRA) under the accession number SRP062395.

### Bacterial community comparison among culture media

We plated the second swab onto four different media types to compare the bacterial isolate richness (alpha-diversity) and community structure (beta-diversity). The culture media types comprised two with a high concentration of nutrients, LB (Luria-Bertani, Fisher Scientific) and TSA (Tryptic soy agar, Remel), and two with a low concentration of nutrients, R2A (Reasoner's 2A, Difco, Becton, Dickinson and Company) and dR2A (1/10 dilution of R2A with an addition of granulated agar so the amount of agar was the same as in the undiluted R2A). We chose commonly used media for our study. For example, LB is often used to culture and maintain *Escherichia coli*, TSA is often used as a non-selective media for general purposes, and R2A, which was initially developed to culture bacteria from potable water (Reasoner and Geldreich, 1985), is the most commonly used media for culturing bacteria from amphibian skin (Harris et al., 2006; Flechas et al., 2012; Antwis et al., 2015; Walke et al., 2015a). For this study, we tested each of the four media types with an aliquot of the TSYE-glycerol solution from each of the 12 toads, resulting in 48 culture plates. We inoculated 30 μl of a briefly vortexed 1:10 dilution of the TSYE-glycerol solutions from the freezer stock of the culture swab onto each media type, followed by spreading the solution across the plate. In addition, a non-inoculated plate for each media type was used as a control to assess the potential for contamination of the media. Following inoculation, all plates were incubated at room temperature in the lab (~24°C).

### Collection of bacterial cultures from plates

After 6 days of incubation, when bacterial colonies started to cover the plates in the high nutrient concentration media, we sampled the entire bacterial community on each plate by applying a slightly modified version of the plate wash PCR procedure (PWPCR) developed by Stevenson et al. (2004). Culture plates were flooded with 3 ml of 1% tryptone broth, and a sterile spreader was used to suspend all visible bacterial colonies. Then the tryptone broth with the suspended bacteria was collected and transferred to a 2 ml sterile collecting tube and centrifuged at 10,000 rpm for 5 min. The resulting supernatant potentially containing bacterially-produced secondary metabolites was filtered through a 0.22 μm filter, and the cell-free supernatant (CFS) was used to conduct *in vitro* challenge assays to determine whether there was community-level variation in the ability to inhibit Bd growth among media types. After removing the supernatant for the challenge assays, we added 2 ml of MicroBead solution (MoBio Laboratories, from Ultraclean Microbial Isolate kit) to each tube of pelleted bacteria and then vortexed to homogenize the solution. These were stored at −20°C prior to DNA extraction. DNA from these cultured bacterial communities was extracted by adding 50 μl of lysozyme solution (20 mg/ml) to each tube followed by an incubation at 56°C for 45 min (Stevenson et al., 2004). After incubation, DNA extractions were performed following the manufacturer's protocol of the UltraClean microbial DNA isolation kit (MoBio Laboratories), which yielded a volume of 100 μl of template DNA.

### Characterization of the cultured bacterial communities

Following DNA extraction, assessment of the cultured communities was done as for the initial culture-independent swab, following methods of Walke et al. (2015a), with the exception that we used 2 μl of DNA template in the PCR. Out of the 48 experimental samples, five (two from the TSA culture media and one from each of the other culture media) were removed from the dataset because they would not amplify. In addition, out of the four control plates, only two could be included because most contained too little DNA for sequencing (as was expected), and even the two that were included had very little DNA. From each of the 45 remaining samples, 200 ng of PCR product was pooled to make a composite sample, which was then cleaned with the QIAquick PCR Purification Kit (Qiagen, Valencia, California). The final pooled sample was sent for sequencing on an Illumina Mi-Seq instrument at the Dana-Farber Cancer Institute of Harvard University following Caporaso et al. (2012) using a 250 bp single-end strategy.

Raw forward 16S rRNA amplicon sequences were demultiplexed and quality-filtered using the default parameters of the Quantitative Insight into Microbial Ecology (QIIME) pipeline (Caporaso et al., 2010b), with a few exceptions: we allowed for no errors in the barcodes, increased the number of minimum consecutive low-quality base calls allowed before truncating a read (r) to 10, and decreased the fraction of the minimum number of consecutive high-quality base calls to include a read (p) to 0.5. Sequences matching PhiX, added to increase base diversity in Illumina sequencing runs, were removed from the dataset using Geneious (Biomatters, Ltd, version 8.1.8). For remaining sequences, a 97% similarity threshold was used to cluster sequences into operational taxonomic units (OTUs, ~bacterial species) using the UCLUST method (Edgar, 2010). Each OTU was represented by the most abundant sequence clustered within it, which was aligned to the Greengenes 13_8 reference database (DeSantis et al., 2006) using PyNAST (Caporaso et al., 2010a) and assigned taxonomy using the RDP classifier (Wang et al., 2007). OTUs assigned to chloroplast or mitochondria were removed, and then OTUs with fewer than 0.01% (524 sequences) of the total reads were removed (Bokulich et al., 2012; Hughey et al., 2016). The sequencing depth per sample ranged from 3,991 to 164,519, including the two controls, which, as expected, had the lowest read counts at 3,991 and 8,299 reads. We removed the controls from the set of samples, given that their removal did not change the total number of OTUs, but would have reduced substantially the cut-off for the standardization of the sampling effort across samples. Thus, we rarefied the sample set to a depth of 59,000 sequences/sample. This final dataset for the culture plates consisted of 347 OTUs across the 43 samples, with 93–277 OTUs per sample (mean ± SD = 174 ± 44). 16S rRNA amplicon sequences are deposited in the NCBI's SRA under the accession number SRP112779.

### Comparison of culture-dependent and culture-independent communities

To determine whether there was variation among media types in the proportion of OTUs obtained from culture plate washes relative to the culture-independent swab from the same individual, we produced a second dataset that incorporated both the amplicon data from the plate washes (*N* = 43) and the culture-independent swabs (*N* = 12). The resulting file was processed in QIIME, as described above, to produce a final OTU table containing all 55 samples. The OTU table was rarefied to 18,000 sequences/sample, which resulted in 459 total OTUs, with 54-254 OTUs per sample (mean ± SD = 148 ± 47). To avoid overestimation in analyses, 139 OTUs associated only with the culture plate samples were removed from the OTU table. These 139 OTUs occurred at very low relative abundances in the culture-independent dataset, and thus were eliminated when filtering out the OTUs below a relative abundance threshold of 0.01 and/or when rarifying the OTU table. By eliminating these OTUs, the dataset contained OTUs present only in the culture-independent swabs or shared between the culture-independent swabs and the culture plates. The final OTU table had a total of 320 OTUs, with 37–220 OTUs per sample (mean ± SD = 106 ± 43).

### Community-level inhibition of Bd growth across individuals and media types

We also assessed whether there was variation among the cultured communities in their ability to inhibit Bd growth. We conducted *in vitro* challenge assays following the method of Bell et al. (2013) and Becker et al. (2015), with the exception that we did not initially co-culture the bacterial isolates with Bd prior to the assay because we were testing whole bacterial communities collected via plate washes. Instead, we used the cell-free supernatant (CFS) that was collected during the plate washes, as described above.

Prior to the challenge assay, zoospores from a Bd culture (JEL404, Maine-USA) were inoculated onto a 1% tryptone agar plate and grown for 3 days at 23°C. The plate was then flooded with 3 ml 1% tryptone broth and the zoospore suspension collected and filtered through a 20 μm filter. The challenge assay was prepared by adding 100 μl of the CFS containing the metabolites from the bacterial communities (*N* = 48 samples; 12 samples/media type) and 100 μl of the Bd zoospore suspension (2 × 10^6^ zoospores per ml) in each well of a sterile 96-well plate. In addition, positive and negative controls were included in each of the 96-well plates. Positive controls were prepared by adding 100 μl of the zoospore suspension and 100 μl of 1% tryptone broth. Negative controls were prepared by adding 100 μl of heat-killed zoospore suspension and 100 μl of 1% tryptone broth. Samples and controls were run in triplicate, and plates were incubated at 23°C. Challenge assay plates were loaded the same day and with the same Bd stock solution. The optical density of each well was measured with a spectrophotometer at 492 nm immediately after plate set up was completed, and then at days 4, 7, and 11. The measurements of optical density were transformed using the formula Ln[OD/(1-OD)]. For each culture plate, the growth rate of Bd in the presence of CFS was calculated by performing a linear regression of the transformed measurements of optical density through time (day 0, 4, 7, and 11). Bd inhibition was calculated by dividing the slope of the triplicates by the average growth rate of the positive control from the respective 96-well plate, and subtracting from 1. Lastly, inhibition values of each triplicate were averaged to calculate the mean inhibition of each culture plate. Negative inhibition values suggest facilitation of Bd growth, while positive values suggest inhibition, with estimates = 1 representing a complete inhibition of Bd growth (Becker et al., 2015).

### Data analysis

Our specific goals were to: (1) compare the diversity of the cultured bacterial communities associated with the different media types representing high and low nutrient concentrations, and identify the cultured bacterial taxa that differed among the media types; (2) determine which media type cultured the highest proportion of OTUs from the full community based on the culture-independent samples; and (3) determine whether the communities growing on the different media types, or from different individual toads, differed in their ability to inhibit Bd growth. Unless noted, all statistical analyses were completed in R version 3.2.4 (R Core Team, 2016). For all generalized linear models (GLMs) and generalized linear mixed models (GLMMs), we performed visual assessments of residual plots with model predictions to confirm that the error distributions used were appropriate.

### Alpha and beta diversity analysis

Alpha diversity estimates were calculated with QIIME for the metrics: richness (OTUs/culture plate), Faith's phylogenetic diversity (measure of diversity based on the branch length of the phylogenetic tree) and the Shannon Index (H', which assesses community evenness). We fitted the diversity metrics to GLMMs. We considered the predictor variable “Media” as a fixed factor and “Individual toad” as a random factor given the nestedness of the media types within individuals. The GLMMs were performed using appropriate error distributions for the diversity metrics to account for heteroscedasticity. For richness, a negative binomial error distribution was applied to the model using the log link function. For phylogenetic diversity and the Shannon index, which was transformed to Hill number (effective number of species; MacArthur, 1965), we used a Gamma error distribution with the inverse link function. The models were run using the R functions glmer.nb for richness, and glmer for phylogenetic diversity and the Shannon Index, from the package *lmer4* (Bates et al., 2014). Multiple comparisons were conducted with Tukey tests using the function glht in the package *multcomp* (Hothorn et al., 2008), which includes multiple comparisons for GLMs. Although not an explicit goal of this study, we also compared the alpha diversity estimates among individual toads since variation in total bacterial diversity across individuals can influence the culturable diversity. For instance, individual toads with high bacterial diversity had higher alpha diversity estimates in their plated communities relative to that from other individuals (Walke et al., 2015a). We used GLMs for this purpose. GLMs were performed as described above, with a negative binomial error distribution for richness estimates and a Gamma error distribution for Shannon and phylogenetic diversity.

Changes in the structure of the bacterial communities across media types were determined by the calculation of dissimilarity distances based on the Bray-Curtis distance measure, which takes into account OTU relative abundance in each community. We are only including the results based on Bray-Curtis since the Jaccard distance measure, which takes into account only the presence/absence composition of the communities, produced consistent results. A statistical comparison of the communities across media types based on the Bray-Curtis dissimilarities was done with a permutational multivariate analysis of variance (PERMANOVA, Anderson, 2001). In addition, we compared the bacterial communities of the media types based on nutrient level, where LB and TSA were grouped as high nutrient concentration media types, and R2A and dR2A as low. When performing the PERMANOVAs, the argument “strata” was used to define the group within which to limit the permutations (i.e., individual toad) due to the nestedness of the replicates of the media types and the potential variation in the skin bacterial communities among toads. Importantly, an analysis excluding those toads that did not have replicates of all media types (toads 4, 11, and 12) was consistent with results from the inclusive analysis that is presented. Lastly, due to potential influence of the individual toads on the plated bacterial communities, as shown in the ordination (**Figure 2**), we also compared the communities across toads where the argument “strata” was not used when performing the PERMANOVAs. Bray-Curtis dissimilarity distances were calculated with the function vegdist, and PERMANOVAs were performed with the function adonis, both functions from the *vegan* package (Okansen et al., 2016). To visualize the results, we used principal coordinate analysis (PCoA).

Reduced variation in community structure across samples could occur on high nutrient media due to fast-growing bacteria out-competing slow-growing bacteria, and among samples from the same individual toad. As a way to test this, we compared the multivariate homogeneity of dispersion (i.e., distance from objects to cluster centroid) across media types based on Bray-Curtis dissimilarity distances. We used the function betadisper from the *vegan* package and conducted a hypothesis test to determine whether there are statistical differences in dispersion among groups using the function anova from the default R package *stats*.

### Species indicator analysis

To identify OTUs associated with particular media types (e.g., TSA vs. LB or low nutrient concentration media vs. high nutrient concentration media), we performed an indicator species analysis using the function multipatt from the *indicspecies* package (De Cáceres and Legendre, 2009). Overall, the function multipatt quantifies, via the estimation of an Indicator Value (IndVal), the association between species (e.g., OTUs) and a group of samples (e.g., replicates within media types) based on the relative abundance and relative frequency of each species, and calculates the statistical significance of the relationships using a permutational approach (De Cáceres and Legendre, 2009).

### Estimating the proportion of OTUs cultured relative to the culture-independent swabs

To account for the individual-level variation in OTU richness, the proportion of OTUs recovered by each media type was calculated at the individual toad level. We then used a GLMM to determine whether media types differed in the number of OTUs cultured relative to the culture-independent swabs. Similar to the alpha diversity analyses, we considered the predictor variable “Media type” as a fixed factor and “Individual toad” as a random factor given the nestedness of the media types within individuals. We used the Gamma error distribution with the inverse link function using the function glmer from the package *lmer4*.

### Comparing community level ability to inhibit Bd growth among culture media types and individual toads

We compared the functional ability to inhibit Bd growth of each bacterial community growing on the culture media plates. We fitted the mean inhibition values to a linear mixed effects model given that the data were normally distributed, as determined by the Lilliefors normality test (function lillie. test from the package *nortest*; Gross and Ligges, 2015). The model included the predictor variable “Media” as a fixed factor and “Individual toad” as a random factor. In addition, to assess the potential effect of individual toads in the inhibition values, we fitted the mean inhibition values to a linear model using the variable “toad” as the predictor variable. The linear mixed effect model was performed using the function lmer from the package *lmer4* (Bates et al., 2014), and the linear model was performed using the function lm from the default R package *stats*.

## Results

**Figure 1:**
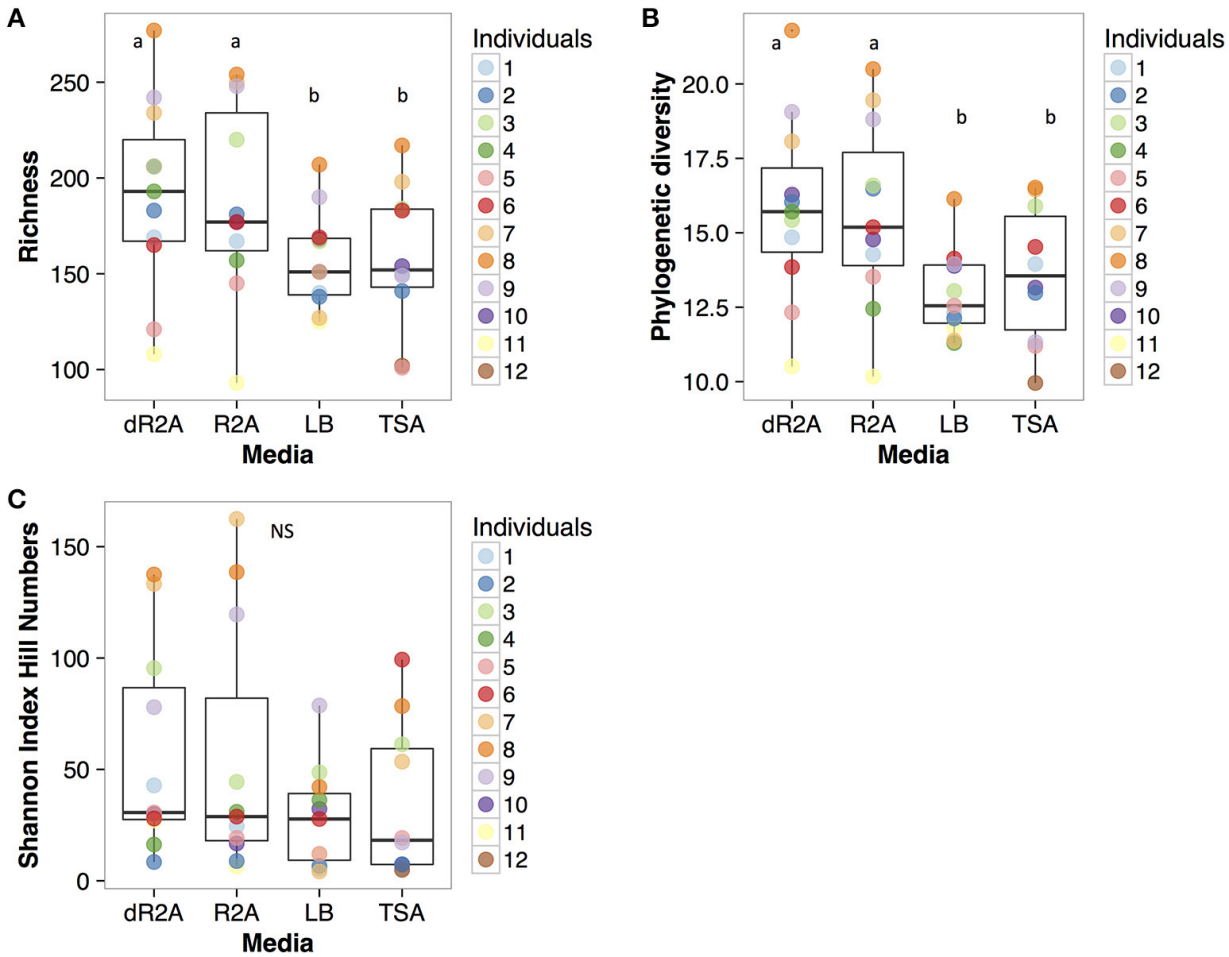
Alpha diversity estimates for the different metrics (**(A)**: OTU richness; **(B)**: Faith's phylogenetic diversity; **(C)**: Shannon Index) by culture media type. Alpha diversity estimates are color-coded at the toad (individual #). Boxplots represent the median, upper and lower quartile, and maximum and minimum values.

Alpha diversity metrics differed significantly among media types for richness and Faith's phylogenetic diversity (richness: Chisq = 15.68, *P* = 0.0013; phylogenetic diversity: Chisq = 25.67, *P* < 0.0001), with the low nutrient concentration media, R2A and dR2A, having a significantly higher diversity compared to the high nutrient concentration media, LB and TSA (Figures 1A,B, Richness: LB-dR2A: *z* = −3.23, *P* = 0.007; TSA-dR2A: *z* = −3.52, *P* = 0.002; R2A-LB: *z* = 2.91, *P* = 0.019; R2A-TSA: *z* = −3.233, *P* = 0.007; Faith's phylogenetic diversity: LB-dR2A: *z* = 4.82, *P* < 0.001; TSA-dR2A: *z* = 3.84, *P* < 0.001; R2A-LB: *z* = −4.58, *P* < 0.001; R2A-TSA: *z* = 3.61, *P* = 0.002). There were no significant differences in the pairwise comparisons between media types within nutrient concentration levels. In contrast to richness and Faith's phylogenetic diversity, we did not find significant differences when comparing the diversity among media types based on Shannon index Hill numbers (Shannon Hill number: Chisq = 7.17, *P* = 0.067), although there was a trend for dR2A and R2A to be higher (Figure 1C). There were also significant differences in the alpha diversity estimates of the plated communities among toads (richness: Deviance = 42.59, *P* < 0.001; phylogenetic diversity: *F statistic* = 3.96, *P* = 0.001; Shannon Hill number: *F statistic* = 6.68, *P* < 0.001).

**Figure 2:**
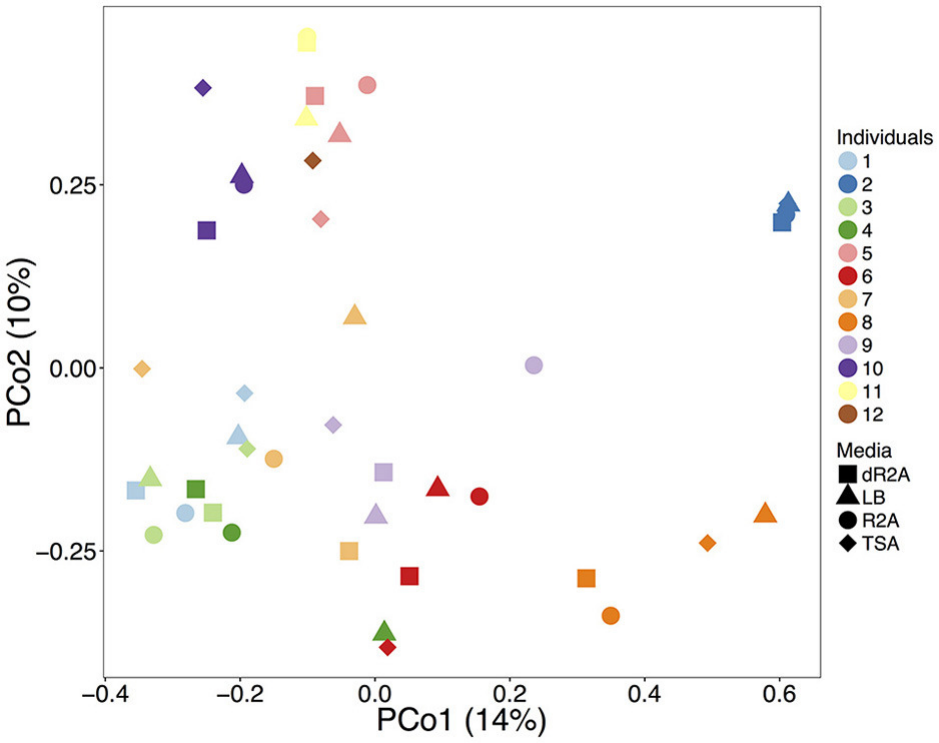
Beta diversity comparisons of the cultured bacterial communities using a principal coordinate analysis (PCoA) ordination based on Bray-Curtis dissimilarity distances. Points represent each plated community. Colors and shapes represent individual toads and culture media types, respectively.

**Figure 3:**
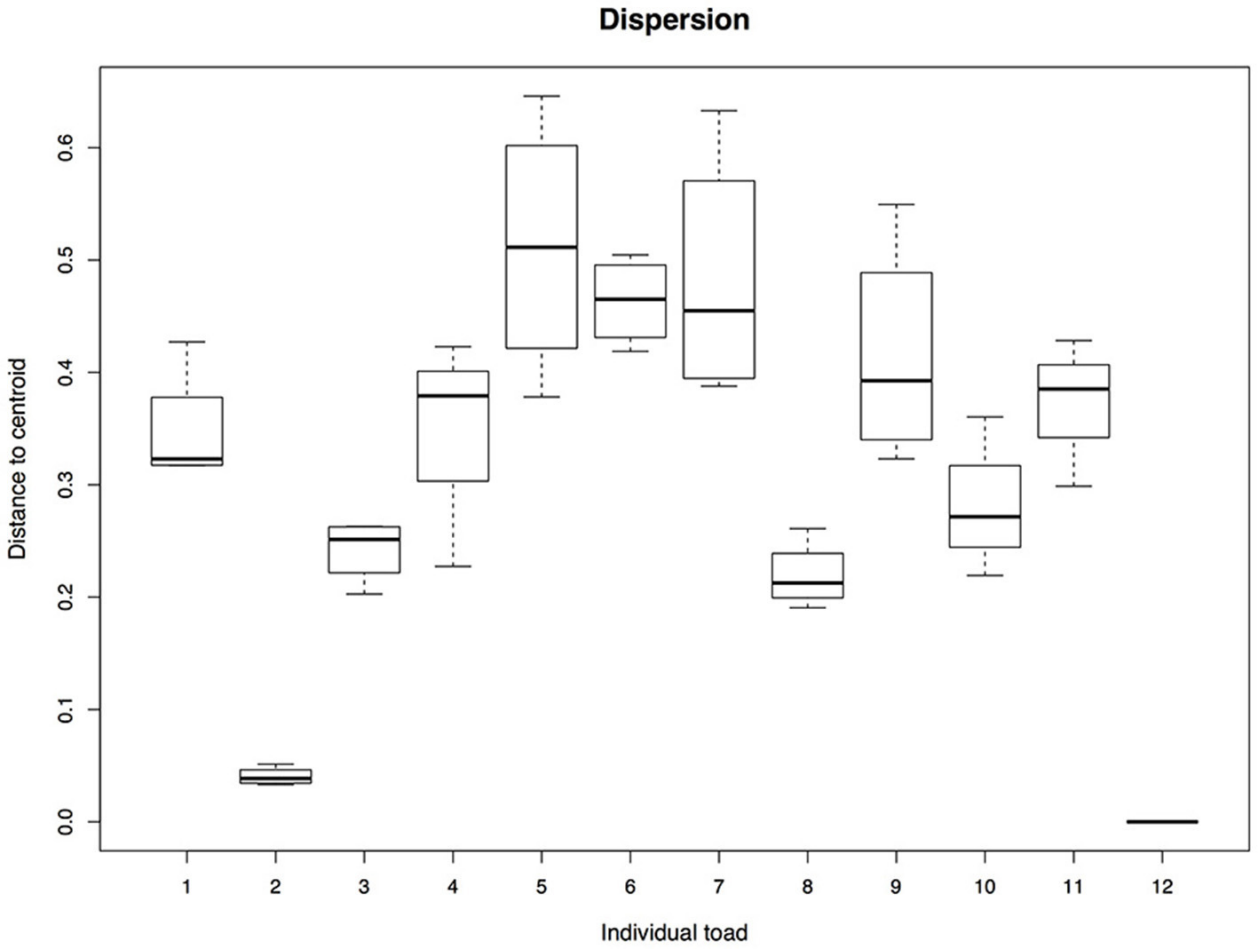
Dispersion among the plated bacterial communities from each individual toad. The analysis was conducted based on Bray-Curtis dissimilarity distances. The figure shows significant differences in dispersion among the individual toads. Boxplots represent the median, upper and lower quartile, and maximum and minimum values.

Bacterial community structure differed among the four media types and also between high and low nutrient concentration media (Figure 2, media types: pseudo-F = 0.68, *R*^2^ = 0.05, *P* = 0.002; nutrient concentration: pseudo-F = 1.14, *R*^2^ = 0.03, *P* < 0.001). However, differences among individual toads explained substantially more variation (pseudo-F = 5.32, *R*^2^ = 0.65, *P* < 0.001). Lastly, the average distances to centroids (~multivariate variance) did not differ significantly among the media types (*F* = 0.83, *P* = 0.48; dR2A 0.57; R2A 0.60; LB 0.61; TSA 0.61). In contrast, distances to centroids did differ among individual toads (Figure 3, *F* = 14.57, *P* = 0.001), which supports the substantial variation explained by individual toads mentioned above, and suggests that individual toads are the main drivers of the observed clustering in Figure 2.

**Figure 4:**
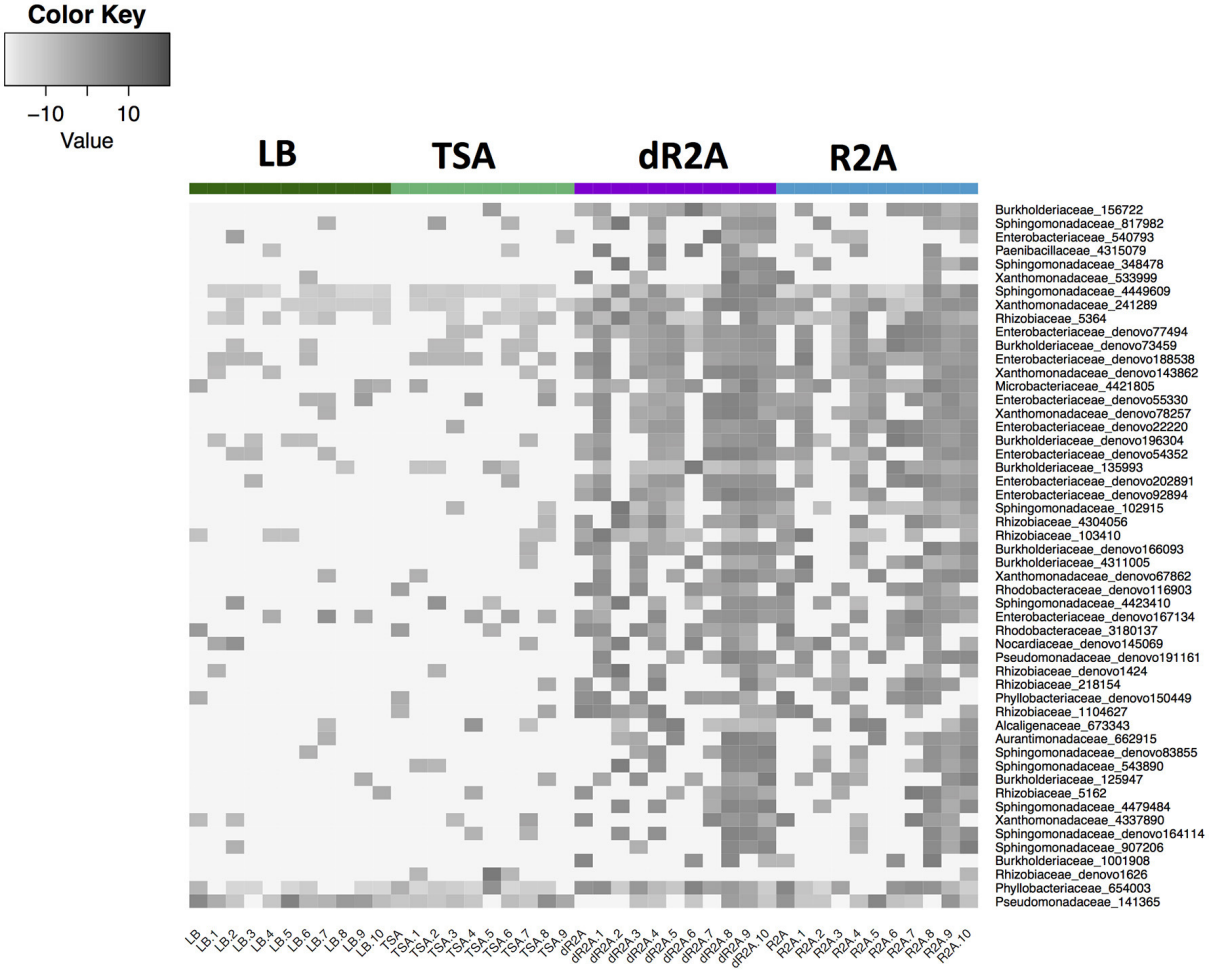
Heatmap of log-transformed relative abundances of the indicator OTUs associated with the different media types (LB, TSA, R2A, and dR2A). Rows indicate each indicator OTU family and ID, and columns indicate the individual toads/replicates within each culture media type.

**Figure 5:**
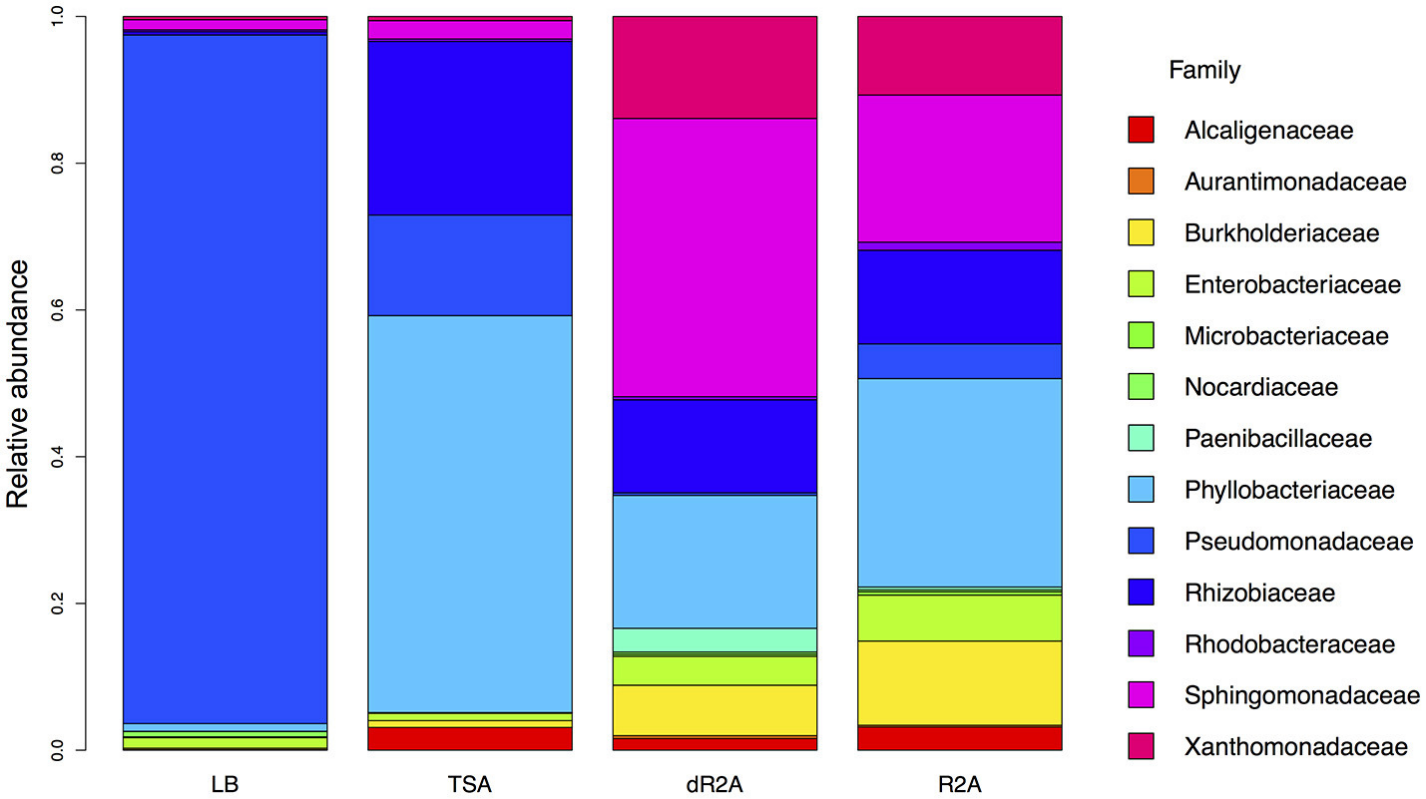
Stacked barplot showing the relative abundance, across toads/replicates within each culture media, of the taxonomic families of the indicator OTUs associated with the distinct culture media types.

**Figure 6:**
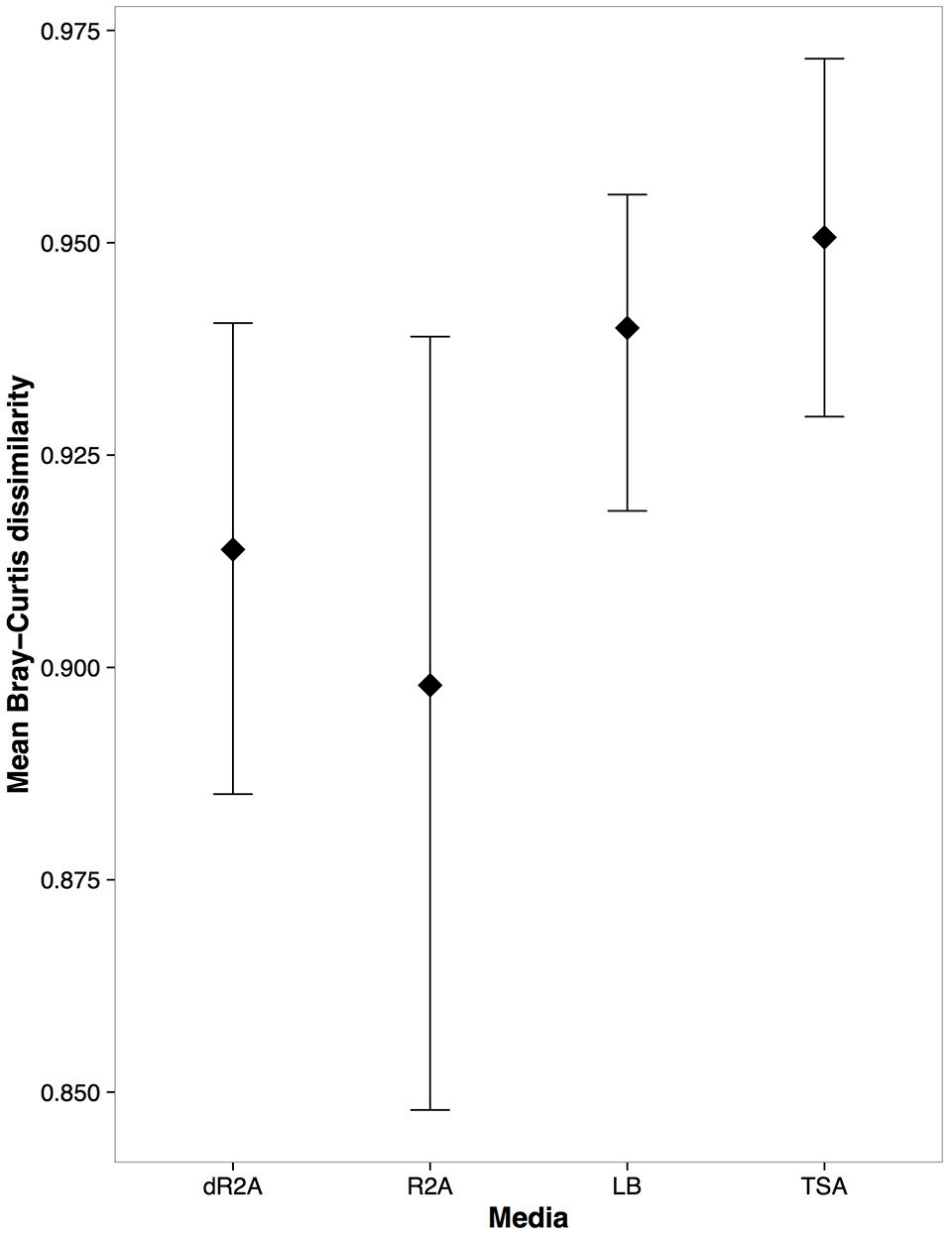
Mean and 95% CI of Bray-Curtis dissimilarity estimates between the cultured communities from the culture media types and the culture-independent bacterial communities from the skin swabs.

We identified a total of 50 indicator OTUs that were significantly (*p* < 0.05) associated with either a specific culture media or high/low nutrient media types. The distribution of these indicator OTUs across media types was: six associated with dR2A, one with TSA, and 41 with the low nutrient media group of R2A and dR2A, suggesting that the low nutrient agars did pick up a unique set of bacteria (Table 1, Figures 4, 5). In addition, there were two indicator OTUs absent in only one of the 4 media types (Table 1): one absent only in LB and another one in dR2A. The relative abundance distribution of the indicator OTUs suggested that some taxa seemed to favor a particular type of culture media. For example, the family *Pseudomonadaceae* had higher relative abundance on LB media, the family *Phyllobacteriaceae* on TSA media, and the families *Sphingomonadaceae* and *Xanthomonadaceae* on the low nutrient media group, R2A and dR2A (Figure 5). Lastly, we also found that a higher proportion of OTUs from the culture-independent swabs were cultured on R2A and dR2A, relative to LB and TSA media plates (Chisq = 26.19, *P* < 0.0001; mean ± sd: R2A 59.65% ± 13.88; dR2A 60.71% ± 14.29; LB 48.47% ± 9.5; TSA 45.38% ± 14.31). This result is further supported by the lower Bray-Curtis dissimilarity distances between the cultured communities and those from the culture-independent swabs (Figure 6).

**Table 1:** List of indicators OTUs associated with the different culture media (LB, TSA, R2A, and dR2A) or group of culture media types.

**Culture media and groups of media**	**Indicator OTU ID**	**Phyla**	**Family**	**Genus**	**Indicator value index**	***P*-value**
dR2A	156722	Proteobacteria	Burkholderiaceae	*Burkholderia*	0.78	0.001
	817982	Proteobacteria	Sphingomonadaceae	*Sphingomonas*	0.50	0.03
	540793	Proteobacteria	Enterobacteriaceae	*Serratia*	0.45	0.047
	4315079	Firmicutes	Paenibacillaceae	*Paenibacillus*	0.43	0.017
	348478	Proteobacteria	Sphingomonadaceae	*Sphingomonas*	0.42	0.022
	533999	Proteobacteria	Xanthomonadaceae	–	0.37	0.039
dR2A + R2A + TSA	654003	Proteobacteria	Phyllobacteriaceae	*Phyllobacterium*	0.90	0.01
LB + R2A + TSA	141365	Proteobacteria	Pseudomonadaceae	*Pseudomonas*	0.86	0.035
R2A + dR2A	4449609	Proteobacteria	Sphingomonadaceae	*Sphingomonas*	0.95	0.01
	241289	Proteobacteria	Xanthomonadaceae	*Luteibacter*	0.91	0.001
	5364	Proteobacteria	Rhizobiaceae	–	0.91	0.002
	denovo77494	Proteobacteria	Enterobacteriaceae	*Erwinia*	0.86	0.001
	denovo73459	Proteobacteria	Burkholderiaceae	*Burkholderia*	0.82	0.001
	denovo188538	Proteobacteria	Enterobacteriaceae	–	0.81	0.003
	denovo143862	Proteobacteria	Xanthomonadaceae	*Luteibacter*	0.77	0.001
	4421805	Actinobacteria	Microbacteriaceae	*Salinibacterium*	0.76	0.002
	denovo55330	Proteobacteria	Enterobacteriaceae	–	0.73	0.004
	denovo78257	Proteobacteria	Xanthomonadaceae	*Luteibacter*	0.73	0.001
	denovo22220	Proteobacteria	Enterobacteriaceae	–	0.73	0.001
	denovo196304	Proteobacteria	Burkholderiaceae	*Burkholderia*	0.72	0.002
	denovo54352	Proteobacteria	Enterobacteriaceae	–	0.72	0.001
	135993	Proteobacteria	Burkholderiaceae	*Burkholderia*	0.72	0.013
	denovo202891	Proteobacteria	Enterobacteriaceae	–	0.72	0.001
	denovo92894	Proteobacteria	Enterobacteriaceae	–	0.68	0.001
	4304056	Proteobacteria	Rhizobiaceae	–	0.68	0.003
	102915	Proteobacteria	Sphingomonadaceae	*Sphingomonas*	0.68	0.012
	103410	Proteobacteria	Rhizobiaceae	*Rhizobium*	0.68	0.031
	denovo166093	Proteobacteria	Burkholderiaceae	–	0.63	0.002
	4311005	Proteobacteria	Burkholderiaceae	*Burkholderia*	0.59	0.007
	denovo67862	Proteobacteria	Xanthomonadaceae	*Luteibacter*	0.59	0.002
	denovo116903	Proteobacteria	Rhodobacteraceae	–	0.58	0.001
	4423410	Proteobacteria	Sphingomonadaceae	*Sphingomonas*	0.58	0.015
	denovo167134	Proteobacteria	Enterobacteriaceae	–	0.57	0.027
	3180137	Proteobacteria	Rhodobacteraceae	–	0.56	0.004
	denovo145069	Actinobacteria	Nocardiaceae	*Rhodococcus*	0.55	0.019
	denovo191161	Proteobacteria	Pseudomonadaceae	*Pseudomonas*	0.55	0.001
	denovo1424	Proteobacteria	Rhizobiaceae	*Rhizobium*	0.54	0.029
	218154	Proteobacteria	Rhizobiaceae	*Agrobacterium*	0.54	0.006
	denovo150449	Proteobacteria	Phyllobacteriaceae	*Phyllobacterium*	0.54	0.003
	1104627	Proteobacteria	Rhizobiaceae	*Rhizobium*	0.54	0.008
	673343	Proteobacteria	Alcaligenaceae	–	0.52	0.027
	662915	Proteobacteria	Aurantimonadaceae	–	0.50	0.009
	denovo83855	Proteobacteria	Sphingomonadaceae	*Sphingomonas*	0.50	0.005
	543890	Proteobacteria	Sphingomonadaceae	*Sphingomonas*	0.50	0.01
	125947	Proteobacteria	Burkholderiaceae	*Burkholderia*	0.50	0.022
	5162	Proteobacteria	Rhizobiaceae	–	0.50	0.036
	4479484	Proteobacteria	Sphingomonadaceae	*Sphingomonas*	0.45	0.006
	4337890	Proteobacteria	Xanthomonadaceae	*Lysobacter*	0.45	0.045
	denovo164114	Proteobacteria	Sphingomonadaceae	*Sphingomonas*	0.41	0.03
TSA	denovo1626	Proteobacteria	Rhizobiaceae	–	0.30	0.032

*Indicator OTUs are listed in a decreasing order based on their indicator value index for a culture media or group of media. The indicator value index represents a measure of the association between an OTU and a culture media or group of media, and range from 0 to 1, where values close to 1 imply a relative stronger association*.

**Figure 7:**
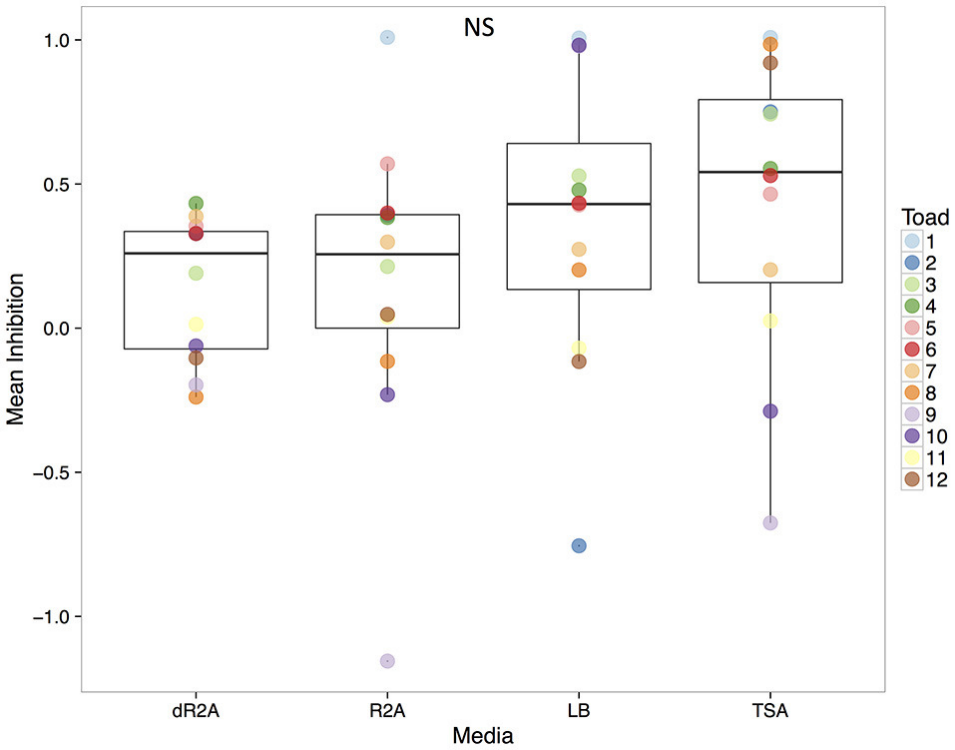
Mean inhibition estimates for each plated community by culture media. Estimates are color-coded at the toad level (individual #). Boxplots represent the median, upper and lower quartile, and maximum and minimum values. Inhibition estimates with negatives values suggest facilitation of Bd growth, while positive values suggest inhibition, with estimates = 1 representing a complete inhibition of Bd growth.

Despite differences across media types in community structure and proportion of recovered OTUs from the culture-independent swabs, we did not find differences among media types in the ability of metabolites from the plated communities to inhibit Bd growth (Figure 7, Chisq = 4.14, *P* = 0.25; mean ± sd: R2A 0.15 ± 0.53; dR2A 0.15 ± 0.25; LB 0.36 ± 0.52; TSA 0.44 ± 0.53). Likewise, despite differences among toads in community structure, we also did not find significant differences in the inhibition values among them (*F* = 1.55, *P* = 0.16). Overall, inhibition estimates across samples ranged from −1.16 (facilitating Bd growth) to 1.01 (completely inhibiting Bd growth), which suggests a substantial amount of variation across samples.

## Discussion

We used amphibian skin bacterial samples to conduct a comparative analysis of the diversity and community structure among plated communities growing on media that varied in nutrient concentration. We addressed the present study from two perspectives: first, from an ecological perspective that aimed to assess how variation in productivity (i.e., distinct nutrient concentrations in the culture media) influences the diversity of the cultured bacterial communities. Second, from an applied perspective that aimed to determine what culture media recovers the highest fraction of the amphibian skin bacterial communities relative to data derived from a culture-independent approach, and to assess whether the plated communities differ in their ability to inhibit the growth of an amphibian pathogenic fungus (Bd strain JEL404). Overall, we found that culture media with low nutrient concentration, R2A and dR2A, facilitated the growth of more diverse and distinct communities relative to the culture media with higher nutrient concentration, LB and TSA. In addition, the high bacterial diversity observed and exclusively associated with R2A and dR2A reflects a higher proportion of the culturable diversity that can be recovered from amphibian skin. However, the estimated function of the plated communities against Bd, based on the whole-plate wash method, did not vary across the culture media or productivity levels. Interestingly, we found that variation among individual hosts influenced the structure of the plated bacterial communities more than the media type. Thus, when attempting to maximize diversity of cultured isolates, swabbing more individuals in the population may be more valuable than using diverse media (Walke et al., 2015a).

The relationship between primary productivity and diversity is considered a key factor determining spatial and temporal diversity patterns in ecological systems (Jessup et al., 2004). Interestingly, in contrast to the general positive quadratic pattern observed in plants and animals (Rosenzweig, 1995), diversity patterns across productivity gradients in microbial systems have been inconsistent across studies (Horner-Devine et al., 2004). The relationship between productivity and diversity in microbial systems has been examined via correlational studies focusing on the variation of bacterial diversity across gradients of nutrient concentration in both laboratory and field studies (Benlloch et al., 1995; Bohannan and Lenski, 2000; Kassen et al., 2000; Horner-Devine et al., 2003). Though our study did not examine the effects of a concentration gradient of a specific nutrient, it did represent microcosms with distinct arrays of nutrients at different concentrations. Moreover, the observed results are consistent with a study showing that diversity patterns in microbial systems can be mediated by the role of ecological factors, such as changes in the relative importance of competition across a gradient of nutrient concentrations (Bohannan and Lenski, 2000). For instance, the observed higher alpha diversity estimates in culture media with low concentrations of nutrients, relative to nutrient-rich ones, highlight the role of competition in plated communities, where faster-growing bacteria tend to outcompete slow-growing bacteria. In addition, culture media with high concentrations of nutrients favoring fast growing bacteria can limit the occurrence of positive relationships between bacterial species, such as cross-feeding and co-aggregation (Vartoukian et al., 2010; Faust and Raes, 2012), which might enhance the growth of slow-growing bacterial taxa.

We observed variation in the structure of the plated communities across media types even though most of the observed variation was driven by differences among individual toads. The observed variation across media types can be explained, in part, due to the fact that culture media types are commonly developed for different purposes and with different degrees of selectivity. For instance, LB and TSA are considered non-selective complex media (i.e., nutrient-rich), whose composition is poorly defined because they usually include complex ingredients, such as yeast extract (Slonczewski and Foster, 2017). In contrast, R2A is considered a defined medium whose chemical components are known (Slonczewski and Foster, 2017) and which selects for slow-growing bacteria, in particular those from aquatic environments (Reasoner and Geldreich, 1985). In the present study, the number and taxonomic classification of the indicator OTUs associated with R2A and dR2A suggests that R2A is the most appropriate media to recover a high diversity of the culturable members of the amphibian skin community. In addition, R2A also recovers some of the most abundant and prevalent taxa occurring on amphibian skin, based on studies of tropical and temperate species (Kueneman et al., 2013; Walke et al., 2014; Becker et al., 2015; Belden et al., 2015; Rebollar et al., 2016b; Medina et al., 2017). For example, we identified several indicator OTUs from the families *Nocardiaceae, Enterobacteriaceae, Pseudomonadaceae, Sphingomonadaceae* and *Xanthomonadaceae* associated with R2A and dR2A that were the most abundant and prevalent bacterial taxa on these individual toads according to 16S rRNA amplicon data (Walke et al., 2015a). Thus, the selection for slow-growing bacteria that characterizes R2A could facilitate the recovery of a higher diversity of culturable bacteria from these communities relative to LB and TSA, which are non-selective complex media and allow faster-growing bacteria to outcompete slow-growing bacteria.

The study by Walke et al. (2015a) that used the same toads aimed to examine the cultured portion of the amphibian skin bacterial communities identified with a culture-independent method, and determined that for each individual toad an average of 0.95% of the community was recovered using R2A. The recovery value estimated by Walke et al. (2015a) is substantially lower compared to that estimated in our study using R2A (i.e., 60%). There are a number of differences in the methods between our study and Walke et al. (2015a) that likely contribute to the observed differences in the number of OTUs recovered. For example, the sampling technique (plate wash in our study vs. pure culture isolation in Walke et al., 2015a) likely influenced the final set of cultured isolates. Pure culture isolation requires manually selecting morphologically-distinct isolates in serial plating, which could result in over-looking related taxa that are morphologically similar, or missing those that require longer incubation periods to have visible colony formation. This could result in an under-estimation of what is cultured. In addition, our present study used a filtering cutoff of 0.01% (maintained only those OTUs with > 0.01% relative abundance), whereas Walke et al. (2015a) used a cutoff of 0.001%. The higher cutoff value 0.01% reduces the number of OTUs in the dataset, which could result in a higher overlap among cultured and culture-independent samples in terms of the OTU composition. We think a cutoff of 0.01%, which was determined in the present study as the cutoff level at which OTU richness leveled off in the dataset (per Bokulich et al., 2012), potentially represents a more realistic view of the actual bacterial species present in the community (Bokulich et al., 2012; Hughey et al., 2016).

Bacterial secondary metabolites produced in response to interspecific interactions have been suggested as a mechanism by which skin bacterial communities can protect their amphibian hosts against Bd infection (Bletz et al., 2013; Walke and Belden, 2016). Furthermore, recent evidence suggests that co-culturing bacterial isolates from amphibian skin can enhance the production of emergent antifungal metabolites, which then have a stronger inhibitory ability against Bd growth relative to monocultures (Loudon et al., 2014; Piovia-Scott et al., 2017). Within the context of our study, even though we observed variation in the diversity of the plated communities across culture media and individual toads, we found no significant differences across these factors in the ability to inhibit Bd growth in challenge assays. In fact, we found a substantial amount of variation, ranging from plated communities that completely inhibited Bd growth to others that enhanced growth. This result is somewhat surprising considering that the cell-free supernatant collected from each of the plated communities should have comprised bacterially-produced secondary metabolites resulting from interspecific interactions. However, given the high diversity of taxa that grew on the plates, variation in density of the distinct bacterial taxa across plated communities, combined with stochastic variation, could have influenced our results (Loudon et al., 2014). Moreover, it is possible that we could have induced additional variation with the method used for collecting the bacterial community and the supernatant from each culture plate (i.e., plate wash; Stevenson et al., 2004). To our knowledge, no previous studies have used the plate wash approach to collect bacterially-produced metabolites. We based this approach on the plate wash method of Stevenson et al. (2004) that was used to survey bacterial communities, as we were interested in trying to get a whole community estimate of potential Bd inhibition. This method was applied under the assumption that anti-fungal metabolites might be produced, at least in part, on the surface of the solid media because the zone of Bd inhibition is commonly observed on the surface of culture plates during Bd challenge assays (Harris et al., 2006; Woodhams et al., 2007; Flechas et al., 2012). Further research will likely be required to fine tune and verify this method as a viable option for assessing whole community Bd inhibition. Our method also did not include the step of co-culturing the plated bacterial communities with Bd, as has been done in previous studies (Becker et al., 2015). However, Becker et al. (2015) found that co-culturing bacterial isolates with Bd had no effect on the ability of bacterial isolates to inhibit Bd growth.

Using culture media with low nutrient concentrations and/or diluted culture media is known to improve the probability of capturing a better representation of bacterial communities living in nature (Vartoukian et al., 2010). The present study provides evidence that low nutrient R2A, relative to other common culture media, allows the growth of a higher diversity of bacterial taxa and recovers a higher proportion of the overall diversity occurring on the amphibian skin. These findings are relevant given that R2A has been widely used in the isolation of amphibian skin bacteria (e.g., Harris et al., 2006; Flechas et al., 2012; Daskin et al., 2014; Shaw et al., 2014; Antwis et al., 2015; Becker et al., 2015; Madison et al., 2017), despite no evidence of its proficiency. We suggest that an understanding of the ecological interactions influencing the plated communities and the nature of the culture media will likely improve our ability to culture rare or previously uncultured microbes. Lastly, we would like to emphasize that, at least in our study species (and see Walke et al., 2015a), swabbing more individuals in a population is the best way to maximize culture collections, regardless of media type. These results can inform current efforts for developing a probiotic-based approach for amphibian conservation, and help to ensure that culture collections are capturing the majority of the important diversity in host-associated microbial systems.

## Author contributions

All authors contributed to the conceptual framework and design of the experiment. DM, ZG, and MS conducted the experiment. DM processed samples in the laboratory. DM, JW, and LB completed data processing and analyses. DM and LB produced the first draft of the manuscript, and all authors edited the manuscript.

### Conflict of interest statement

The authors declare that the research was conducted in the absence of any commercial or financial relationships that could be construed as a potential conflict of interest.
